# Medication-Related Burden and Medication Adherence Among Geriatric Patients in Kuwait: A Cross-Sectional Study

**DOI:** 10.3389/fphar.2020.01296

**Published:** 2020-08-21

**Authors:** Abdelmoneim Awad, Anwar Alhadab, Abdullah Albassam

**Affiliations:** Department of Pharmacy Practice, Faculty of Pharmacy, Kuwait University, Kuwait City, Kuwait

**Keywords:** medicine burden, medication-related burden, medication adherence, geriatrics, patient experience, Kuwait

## Abstract

The evaluation of medicines burden from the patients’ perspectives is a crucial endeavor to identify any barriers that may hinder achieving optimal health outcomes. Therefore, this study was designed, firstly to identify the prevalence of medication-related burden among geriatrics and factors influencing this burden. Secondly, to determine the prevalence of medication adherence and the correlation between the burden and adherence. A cross-sectional study was performed using Living with Medicines Questionnaire version-3 (LMQ-3) and Adherence to Refills and Medications Scale (ARMS) questionnaire. Four hundred and fifty patients attending primary healthcare centers were invited to participate, and 424 (94.2%) agreed. Data were collected *via* face-to-face structured interviews. The vast majority of respondents (97.4%; 95% CI: 95.3–98.6) perceived to suffer from minimum (35.4%) to moderate (62.0%) degrees of medicine burden. The median (IQR) LMQ overall score was 112 (21) indicating a moderate burden. LMQ-3 overall scores revealed a significant trend toward higher perceived burden among respondents aged ≥ 75 years, males, non-Kuwaitis, residents in Al-Farwaniyah and Al-Jahra governorates, using oral and nonoral formulations, paying prescription charges, and needing support with using medicines (p <0.05). Almost 55% (95% CI: 49.8–59.5) of respondents were nonadherent to their medications. The median (IQR) ARMS overall score was 20 (7.0) indicating low adherence to medications. There was a significant positive correlation between LMQ-3 and ARMS scores (p<0.001) showing that the higher the medications burden the lower the level of medication adherence. The key findings of this study underscore the need for multifaceted interventions that could be targeted at the identified problems to reduce medication burden and improve medication adherence.

## Introduction

The burden of noncommunicable chronic diseases (NCDs) is rapidly increasing globally. In 2018, the World Health Organization (WHO) reported that NCDs are accountable for 41 million deaths annually, which is equal to 71% of global deaths. The four major chronic diseases accounting for 79% of NCD deaths, include, cardiovascular diseases, cancers, respiratory diseases, and diabetes (WHO, 2018). In Kuwait, NCDs are the major cause of deaths among the geriatric population (Younis et al., 2015). Despite global efforts to reduce the burden of these NCDs (WHO, 2013), the major emphasis of the clinical practice guidelines and healthcare practitioners is to achieve the desired clinical therapeutic outcomes for each NCD that may lead to better disease management, but also may generate a drive toward prescribing multiple medications that are becoming burdensome to patients (May et al., 2009; Eton et al., 2012). Attention to patients’ experiences with medicines is not optimal in clinical practice and clinicians are often unaware of the challenges patients face with medicines (Tran et al., 2014). It was reported that patients with multiple NCDs are often exposed to a fragmented care approach, and trying to cope with various recommendations and complex medication therapy regimens that adversely affect their quality of life (QoL) (Tinetti and Fried, 2004; Boyd et al., 2005).

The prevalence of prescribing multiple medications to patients is increasing globally due to the rise in life expectancy and the incidence of multiple NCDs (Barrett et al., 2016). Geriatric patients are more prone to polypharmacy, which is associated with medication-related adverse events along with drug-disease and/or drug-drug interactions, and nonadherence that may result in a sub-therapeutic effect, increased hospitalization, falls, cognitive impairment, and increased healthcare expenditures (Guthrie et al., 2015; Morgan et al., 2016; Dhalwani et al., 2017). In Kuwait, a study performed among elderly patients in primary healthcare centers identified that 72.1% were using ≥ 5 medications, 69.5% had ≥ 3 NCDs, and over 50% were taking potentially inappropriate medications that may increase the risk of adverse clinical outcomes among these patients (Awad and Hanna, 2019). Most of the published literature interpreted the biomedical perspective of medication-related burden (MRB) as the number of medications or pills taken daily by individual patients for the treatment of their diseases, disregarding the patient’s crucial perspectives (Krska et al., 2014; Krska et al., 2019). Published systematic reviews revealed that several patients had negative views toward medicines, with some refusing to use medications due to their concerns about adverse effects or dependence. It was found that MRB plays a pivotal role in affecting patients’ health, well-being, beliefs and behavior toward medications (Pound et al., 2005; Mohammed et al., 2016). An Australian study among elderly patients revealed that over 60% reported that they were using a large number of medications and 92% indicated their willingness to stop using one or more (Reeve et al., 2013). It was reported that several geriatric patients’ lives are governed by the medicine regimens they must follow. They experience varying degrees of difficulty in complying, which adversely affected their QoL (Eastleigh Southern Parishes Older People’s Forum, 2008).

Evidence has shown that using multiple medicines for NCDs can be burdensome to patients and this burden is influenced by numerous factors such as medication regimens, formulations, side effects, experiences with healthcare, and social burden (Krska et al., 2013; Krska et al., 2018). Furthermore, it was found that healthcare practitioners give less attention to patients’ psychosocial information compared to biomedical factors in making therapeutic decisions (Weiner et al., 2010). International clinical guidelines advocate patient-centered care that necessitates the clinicians to determine patients’ experiences of using medications (Royal Pharmaceutical Society, 2013; National Institute for Health and Care Excellence, 2017). Thus, there is a need for healthcare practitioners to ensure patients’ involvement in initial prescribing decisions, listening to and having insight into their lived experiences with medications including any difficulties and concerns in medication therapy plans (Royal Pharmaceutical Society, 2013; Mohammed et al., 2016). It was reported that positive experiences with medications such as good relationships with clinicians, clear benefits of the medications, and ease of maintaining medicines routine resulted in improving control of patients’ conditions and clinical indicators, while negative experiences can lead to adverse events, poor disease control, inconvenience or inappropriate use of medicine, and poor QoL (Krska et al., 2013; Mohammed et al., 2016). The experience of patients taking long-term medicines as a key element of quality healthcare is worth to be studied in order to enhance successful therapeutic decisions. There is a recent increase in the number of studies investigating MRB from the patients’ perspectives (Mohammed et al., 2016). MRB is a great component of overall treatment burden, and because medications are the major means of managing most diseases, they are the most frequently identified part of treatment burden in qualitative studies (Salahudeen et al., 2015). Due to the extent of medications usage as treatment options, patients’ perspectives of medication burden merits distinct exploration from the treatment burden, and this should focus solely on medicine use (Krska et al., 2019). Most of the published qualitative and quantitative research studies related to patient’s experience with medicines have centered on particular aspects such as the capacity to manage medications, satisfaction with medications, knowledge, and beliefs about medications, and medication-taking behaviors with or without considering particular medication therapies or medical conditions (Atkinson et al., 2004; Pound et al., 2005; Elliott and Marriott, 2009; Salahudeen et al., 2015; Mohammed et al., 2016). However, there is limited research work to develop tools to evaluate various aspects of medications use from the patients’ perspectives to quantify the overall burden of using medications. One of these developed tools is the validated Living with Medicines Questionnaire version 3 (LMQ-3), which unlike most other patient-reported measures, was developed from the patients’ perspectives and included eight domains that could be burdensome to patients. It quantifies the types of a wide range of issues patients experience with medications that contribute to overall MRB. Also, it identifies patients at a higher risk of issues related to MRB who could benefit from interventions (Katusiime et al., 2018). Few published studies assessed multiple aspects to quantify overall MRB from the patient perspective using the LMQ-3 (Katusiime et al., 2018; Krska et al., 2018; Zidan et al., 2018; Krska et al., 2019).

The elderly population in Kuwait accounts for 2.33% of the total population. This is expected to increase to 4.41% and 17.9% by 2025 and 2050, respectively (World Population Review, 2020). Due to the increase in this population, it is a vital requirement to promote the optimal prescribing of medications and improve their health and QoL. Hence, the evaluation of the MRB from the geriatric patients’ perspectives is a crucial endeavor to identify any barriers that may hinder achieving optimal health outcomes. To the best of our knowledge, there is only one published study that quantitatively assessed the overall MRB from the patient’s perspective in the Middle East and North Africa (MENA) region. It was conducted in Qatar among adult patients with diabetes, with or without other comorbidities (Zidan et al., 2018). However, there are no similar published studies to date in Kuwait. Thus, this study was designed to measure the issues geriatric patients experience with medications that contribute to the overall burden as well as the factors that influence MRB. In addition, it determined the prevalence of self-reported adherence to medications and the correlation between the self-reported MRB and the self‐reported medication adherence (MA).

## Materials and Methods

### Study Area

Kuwait is a Middle-Eastern country with an area of 17,820 km^2^ and an estimated total population of 4,267,827 individuals (2019 estimate) (World Population Review, 2020). In Kuwait, the healthcare system includes public and a private sector. The public sector is the largest provider of healthcare providing comprehensive advanced health services free of charge for the Kuwaiti nationals. A public insurance scheme exists to provide the same services for the non-Kuwaiti residents at a reduced cost. The public sector comprises of primary, secondary, and tertiary levels of healthcare delivery. Primary care is delivered through healthcare centers distributed over the six governorates of Kuwait. Secondary healthcare services are offered by general public hospitals. Tertiary health care services are provided through specialized healthcare facilities that focus on specific health conditions.

### Study Design and Population

A descriptive and cross-sectional survey-based study was conducted in 10 primary healthcare centers that provide geriatric care through specialized geriatric clinics. The target population was geriatric patients, aged ≥ 65 years using at least one prescribed medicine for any chronic disease/condition. Patients with psychiatric disorders or cognitive impairment were excluded from the study. Ethical clearance was obtained from the Ministry of Health Ethical Committee, Kuwait. The sample size was determined on the assumption that the percentage of patients suffering from MRB is 50% since there are no previous similar reports from Kuwait. It is computed using the Raosoft sample size calculator using a margin of error of 5%, a confidence interval of 95%, a population size of 96,600 people aged ≥ 65 years old (Raosoft, 2004). The minimum sample size was determined to be 382. Assuming a response rate of 85%, a larger sample size of 450 patients were invited to participate in this study.

A list of the primary healthcare centers that provide geriatric care through specialized geriatric clinics at the time of the study was obtained from the Ministry of Health. It included 18 geriatric clinics distributed among six governorates. Stratified random sampling was used to select 10 healthcare centers from the six governorates. The total number of geriatric patients’ files at each of the selected healthcare centers was obtained, and stratified random sampling was used to determine the number of patients that should be invited to participate in the study from each healthcare center. The patients who attended these healthcare centers during the days for data collection were selected using systematic random sampling.

### Study Questionnaire

The study questionnaire included four parts: demographic characteristics, questions about medicines, LMQ-3, and Adherence to Refills and Medication Scale (ARMS) questionnaire. The first part consisted of six questions related to age, gender, nationality, educational level, residence, and employment status. Part two contained five questions related to the number of regularly used prescription medicines, types of formulations, frequency of dose, payment for a prescription charge, and need for support with using medicines. The third part covered a 41-item questionnaire (LMQ-3) in which the participants indicated their level of agreement using a five-point Likert scale (from 1=strongly agree to 5=strongly disagree). An open-ended question was also included to allow the participants to provide any other relevant issues that are not covered in the tool. It consisted of eight domains: relationships/communication with healthcare professionals about medicines (five items), practical difficulties (seven items), cost-related burden (three items), side-effects burden of prescribed medications (four items), perceived effectiveness of medicines (six items), attitudes/concerns about medicine use (seven items), interferences with day-to-day life (six items), and control/autonomy of medicine use (three items). Domain scores are summed to produce a total scale score (LMQ‐3 overall score) illustrating the overall level of MRB ranged from 41 to 205, with higher scores indicating higher MRB. It also contained a 10-cm visual analog scale ranging from 0 “no burden at all” to 10”extremely burdensome” allows self‐reporting of overall perceived burden (VAS‐burden). The LMQ-3 was validated in English (Katusiime et al., 2018), and was also adapted and validated in the Arabic language (Zidan et al., 2016). Part four included a 12-item questionnaire (ARMS). The 12 items included 8 items to assess the degree to which patients took their medication as directed and 4 items related to the prescription refill to assess the refill adherence. Each item was rated using a four-point Likert scale, ranging from 1 (none of the time) to 4 (all of the time). The overall ARMS score ranged from 12 to 48, with lower scores indicating better adherence. The scale showed high internal consistency reliability and a significant correlation with the Morisky Adherence Scale (Kripalani et al., 2009). It had also been translated into the Arabic language (Zidan et al., 2018). The Arabic versions of the LMQ-3 and ARMS were used in this study. The study questionnaire was piloted with 10 geriatric patients for content, design, and comprehension, and no amendments were made. The items of the study survey and their quantified responses are shown in tables in the results. Permission was obtained from the original developers of the questionnaires and the authors of the translated Arabic versions to be used in the present study.

### Data Collection

Data were collected prospectively *via* face-to-face structured interviews of the study participants in the waiting areas of the geriatric clinics using the piloted questionnaire. Patients who agreed to participate in the study were assured of the confidentiality and gave written consent to participate in the study.

### Statistical Analysis

Data were entered into the Statistical Package for Social Sciences (IBM SPSS Statistics for Windows, version 25), descriptive and comparative analysis was conducted. The results were presented as percentages (95% Confidence interval-CI), medians (interquartile range-IQR), means (standard deviation-SD), and range. Respondents’ experiences with medication use are presented as percentages of participants who strongly agreed/agreed, had neutral responses, and strongly disagreed/disagreed with each item. The LMQ overall score was calculated as the sum of the scores of all of the 41 items in the survey and ranged from 41 to 205. The negatively worded statements were reverse coded for calculation of the score. The degree of MRB is categorized as follows based on the LMQ overall score: (1) no burden at all (41-73); (2) minimum burden (74-106); (3) moderate burden (107-139); (4) high burden (140-172); and (5) Extremely high burden (173-205) (Zidan et al., 2018). The overall ARMS score was calculated as the sum of the points for the 12 items and it ranged from 12 to 48. Item number 12 in the ARMS scale was negatively worded and was reverse coded for calculation of the score. The overall ARMS score was dichotomized based on a cutoff value of median score 20 (low adherence ≥ 20 and high adherence < 20) (Jin et al., 2016). Comparative analysis was conducted to the MRB (LMQ-3 individual scores and overall scores) across different demographic and medicine-related characteristics of the respondents using Mann‐Whitney and Kruskal‐Wallis tests because the data were not normally distributed based on the Shapiro-Wilk and Kolmogorov-Smirnov tests for normality. Statistical significance was accepted at p < 0.05. The demographic and medicine-related characteristics of the respondents were categorized as follows: (1) age: 65–74 years and ≥75 years; (2) gender: male and female; (3) nationality:Kuwaiti nationals and non-Kuwaiti residents; (4) level of education: low-intermediate [0–12 years] for those who completed secondary school or less and high [>12 years] for those who had a diploma, or bachelor degree or postgraduate degree; (5) area of residence: Capital, Hawalli, Al-Farwaniyah, Al-Ahmadi, Al-Jahra, and Mubarak Al-Kabeer; (6) employment status: employed and unemployed/retired; (7) number of medications: 1–4 medications and ≥ 5 medications; (8) formulations used: oral solid dose formulations and combination of oral solid dose and nonoral formulations; (9) frequency of dose: once daily, twice daily, three times daily, and > 3 times daily; (10) paying for prescription charges: yes and no; and (11) need help with using medicines: yes and no. Spearman rank correlation was also used to analyze the correlation between the perceived MRB and MA.

## Results

### Demographic and Medication-Related Characteristics of the Respondents

A total of 450 geriatric patients were approached to be included in the study, 424 agreed to participate giving a response rate of 94.2%. Their median (IQR) age was 70 (8) years [mean (SD) 71.4 (5.0); range 65–88). Of the 114 patients aged ≥75 years, 6 (5.3%) of them were ≥85 years old. The median (IQR) number of medications per patient was 5.0 (3.0) [mean (SD) 4.9 (2.4); range 1–15). Of the 230 patients prescribed ≥ 5 medications, 19 (8.3%) of them were prescribed ≥10 medications. Over three-quarters of the participants were using oral solid dose formulations (tablets and/or capsules) and were taking their medications two or three times daily. Most of the patients were not paying for their prescribed medications and over two-thirds indicated that they were not receiving help with using their medicines. **Table 1** shows demographic and medication-related characteristics of the respondents.

**Table 1:** Demographic and medication-related characteristics of the respondents (n = 424).

Characteristics	Frequency (%)
**Demographic characteristics**	
**Age (Years)** 65–74 ≥ 75	310 (73.1) 114 (26.9)
**Gender** Male Female	200 (47.2) 224 (52.8)
**Nationality** Kuwaiti national Non-Kuwaiti resident	406 (95.8) 18 (4.2)
**Education level** Low-Intermediate Education High Education	257 (60.6) 167 (39.4)
**Residence** Capital Hawalli Al-Farwaniyah Al-Ahmadi Al-Jahra Mubarak Al-Kabeer	71 (16.7) 82 (19.3) 88 (20.8) 113 (26.7) 30 (7.1) 40 (9.4)
**Employment status** Employed Unemployed/Retired	48 (11.3) 376 (88.7)
**Medication-related Characteristics**	
**Number of Medications** 1–4 ≥ 5	194 (45.8) 230 (54.2)
**Formulation used** Tablets/capsules Tablets/capsules + nonoral	325 (76.7) 99 (23.3)
**Frequency of daily dose** Once Twice Three times More than three times	41 (9.7) 134 (31.6) 199 (46.9) 50 (11.8)
**Paying for prescriptions** No Yes	406 (95.8) 18 (4.2)
**Needing help with using medicines** No Yes **If yes, (n=127)** Husband/Wife Relative Nurse Housemaid	297 (70.0) 127 (30.0) 23 (15.2) 44 (40.9) 40 (22.7) 20 (21.2)

### Assessment of Medication-Related Burden Using LMQ-3 and Adherence to Medications Using ARMS

**Table 2** presents the responses to individual items within the eight domains of LMQ‐3. Demographic and medication-related characteristics affected scores across all domains. Educational level and employment status affected one domain; age and gender affected two domains; nationality, frequency of dose, and paying a prescription charge affected three domains; the number of medicines and types of formulations affected four domains, and needing assistance with use of medicines affected seven domains (**Table 3**). In relation to communication/relationships with healthcare professionals about medicines, 66.7% (95% CI: 62.0–71.2) of respondents trusted the judgment of their doctors in choosing medicines for them. About three-fifths judged that doctors listen to their opinions (59.0%; 95% CI: 54.1–63.7), take their concerns about side effects seriously (58.5%; 95% CI: 53.6–63.2), get enough information about medicines from their doctors (60.6%; 95% CI: 55.8–65.3), and that healthcare professionals know enough about them and their medicines (60.8%; 95% CI: 56.0–65.5). In this domain, significantly higher scores indicating poorer quality relationships were found among respondents aged ≥ 75 years, males, using oral solid dose and nonoral formulations, taking medicines more than three times daily, and needing help with using medicines (**Table 3**).

**Table 2:** Responses to individual items in the eight domains of the Living with Medicines Questionnaire version-3 (LMQ‐3) (n=424).

Statement	Strongly agreed/agreed n (%)	Strongly disagreed/disagreed n (%)	Neutral n (%)
**Theme 1: Relationships/communication with healthcare professionals about medicines**			
I trust the judgment of my doctor(s) in choosing medicines for me.	283 (66.7)	57 (13.4)	84 (19.8)
My doctor(s) listen to my opinions about my medicines.	250 (59.0)	75 (17.7)	99 (23.3)
My doctor(s) takes my concerns about side effects seriously.	248 (58.5)	79 (18.6)	97 (22.9)
I get enough information about my medicines from my doctor(s).	257 (60.6)	84 (19.8)	83 (19.6)
The health professionals providing my care know enough about me and my medicines.	258 (60.8)	78 (18.4)	88 (20.8)
**Theme 2: Practical Difficulties**			
I find getting my prescriptions from the doctor difficult.*	84 (19.8)	300 (70.8)	40 (9.4)
I find getting my medicines from the pharmacist difficult.*	60 (14.2)	321 (75.7)	43 (10.1)
I am comfortable with the times I should take my medicines.	305 (71.9)	78 (18.4)	41 (9.7)
I am concerned that I may forget to take my medicines.*	293 (69.1)	94 (22.2)	37 (8.7)
I have to put a lot of planning and thought into taking my medicines.*	155 (36.5)	181 (42.7)	88 (20.8)
It is easy to keep to my medicines routine.	270 (63.7)	98 (23.1)	56 (13.2)
I find using my medicines difficult.*	104 (24.5)	228 (53.8)	92 (21.7)
**Theme 3: Cost-related burden**			
I worry about paying for my medicines.*	7 (1.6)	412 (97.2)	5 (1.2)
I sometimes have to choose between buying basic essentials or medicines.*	6 (1.4)	415 (97.9)	3 (0.7)
I have to pay more than I can afford for my medicines.*	4 (0.9)	416 (98.1)	4 (0.9)
**Theme 4: Side-effects burden of prescribed medications**			
The side effects I get are sometimes worse than the problem for which I take medicines.*	220 (51.9)	112 (26.4)	92 (21.7)
The side effects I get from my medicines interfere with my day-to‐day life (e.g. work, housework, sleep).*	179 (42.2)	155 (36.6)	90 (21.2)
The side effects I get from my medicines are bothersome.*	180 (42.5)	138 (32.5)	106 (25.0)
The side effects I get from my medicines adversely affect my well‐being.*	196 (46.3)	129 (30.4)	99 (23.3)
**Theme 5: Perceived effectiveness of medicines**			
I am satisfied with the effectiveness of my medicines.	285 (67.2)	70 (16.5)	69 (16.3)
My medicines prevent my condition getting worse.	310 (73.1)	46 (10.9)	68 (16.0)
My medicines live up to my expectations.	297 (70.0)	52 (12.3)	75 (17.7)
My medicines allow me to live my life as I want to.	292 (68.9)	70 (16.5)	62 (14.6)
My medicines are working.	299 (70.5)	37 (8.7)	88 (20.8)
The side effects are worth it for the benefits I get from my medicines.	284 (67.0)	45 (10.6)	95 (22.4)
**Theme 6: Attitudes/concerns about medicine use**			
I worry that I have to take several medicines at the same time.*	241 (56.8)	145 (34.2)	38 (9.0)
I would like more say in the brands of medicines I use*	107 (25.2)	197 (46.5)	120 (28.3)
I feel I need more information about my medicines*	248 (58.5)	117 (27.6)	59 (13.9)
I am concerned about possible damaging long term effects of taking medicine*	252 (59.4)	103 (24.3)	69 (16.3)
I am concerned that I am too reliant on my medicines*	224 (52.8)	139 (32.8)	61 (14.4)
I am concerned that my medicines interact with my Nutritional habits.*	231 (54.5)	121 (28.5)	72 (17.0)
I worry that my medicines may interact with each other.*	243 (57.3)	113 (26.7)	68 (16.0)
**Theme 7: Interferences with day-to-day life**			
My medicines interfere with my social or leisure activities.*	173 (40.8)	188 (44.3)	63 (14.9)
Taking medicines affects my driving.*	94 (22.2)	224 (52.8)	106 (25.0)
My medicines interfere with my social relationships.*	118 (27.8)	228 (53.8)	78 (18.4)
Taking medicines causes me problems with daily tasks such as (work, housework, hobbies).*	141 (33.3)	196 (46.2)	87 (20.5)
My medicines interfere with my sexual life.*	75 (17.7)	227 (53.5)	122 (28.8)
My life revolves around using my medicines.*	244 (57.5)	86 (20.3)	94 (22.2)
**Theme 8: Control/autonomy of medicine use**			
I can vary the dose of the medicines I take.	133 (31.4)	217 (51.2)	74 (17.4)
I can choose whether or not to take my medicines.	115 (27.1)	213 (50.3)	96 (22.6)
I can vary the times I take my medicines.	139 (32.8)	222 (52.4)	63 (14.8)

*Negatively worded statements were reverse coded for calculation of the score.

**Table 3:** Effect of demographic and medication-related characteristics of respondents on individual domains of Living with Medicines Questionnaire version-3 (LMQ‐3) (n=424).

Characteristics	Median (IQR) domain score (^Maximum possible score)							
	Relationships (25)	Practicalities (35)	Cost (15)	Side effects (20)	Effectiveness (30)	Concerns (35)	Interference (30)	Autonomy (15)
**Age (Years)** 65-74 ≥ 75	11 (6) 12.5 (5)	18 (5) 18.5 (5)	3 (0) 3 (0)	12 (5) 14 (5)	12 (6) 14 (6)	24 (9) 24 (7)	17 (6) 17 (5.3)	10 (4) 10 (4)
*p*-value	0.009*	0.338	0.867	0.084	0.024*	0.531	0.346	0.992
**Gender** Male Female	13 (6.8) 10 (6)	19 (4) 18 (5)	3 (0) 3 (0)	13 (6) 13 (4)	14 (5) 12 (6)	24 (8) 24 (7)	17 (6) 17 (5.8)	10 (4) 10 (4)
*p*-value	<0.001*	0.071	0.867	0.442	<0.001*	0.851	0.630	0.856
**Nationality** Kuwaiti nationals Non-Kuwaiti residents	11 (7) 13.5 (5.8)	18 (5) 19 (6.3)	3 (0) 8.5 (5.5)	13 (4.3) 13.5 (4.3)	12 (6) 12 (5)	24 (7.3) 26.5 (8)	17 (5.3) 20 (6.3)	10 (4) 10.5 (3.3)
*p*-value	0.140	0.114	<0.001*	0.175	0.387	0.033*	0.017*	0.481
**Education level** Low-Intermediate High	12 (7) 11 (5)	18 (5) 18 (5)	3 (0) 3 (0)	13 (5) 12 (4)	13 (6) 12 (6)	24 (9) 24 (7)	17 (6) 17 (5)	10 (4) 10 (4)
*p*-value	0.670	0.497	0.877	0.035*	0.343	0.671	0.542	0.886
**Employment status** Employed Unemployed/Retired	12 (6.8) 11 (7)	19 (5) 18 (5)	3 (0) 3 (0)	12 (4) 13 94)	14 (7.5) 12 96)	24 (9.8) 24 (7)	16.5 (5.8) 17 (6)	10 (4) 10 (4)
*p*-value	0.241	0.261	0.877	0.077	0.036*	0.764	0.361	0.630
**Medication-related**								
**Number of Medications** 1-4 ≥ 5	11 (7) 12 (6)	18 (5) 18 (5)	3 (0) 3 (0)	12 (5) 13 (4.3)	12 (7) 13 (4.3)	23 (7.5) 24 (7.3)	16 (6) 18 (6)	10 (4) 11 (6)
*p*-value	0.114	0.686	0.882	0.014*	0.914	0.001*	0.030*	0.041*
**Formulation used** Tablets/capsules Tablets/capsules + nonoral	11 (6) 13 (6)	18 (5) 19 (5)	3 (0) 3 (0)	12 (4.5) 13 (5)	12 (5) 15 (6)	24 (7) 24 (8)	16 (6.5) 18 (5)	10 (4) 10 (4)
*p*-value	0.004*	0.016*	0.678	0.252	<0.001*	0.359	0.009*	0.189
**Frequency of dose/day** Once Twice Three times More than three times	11 (5.5) 12 (5.3) 11 (7) 14 (7.3)	17 (6.5) 18 (5) 19 (4) 18.5 (5)	3 (0) 3 (0) 3 (0) 3 (0)	12 (4) 12 (5) 13 (5) 12 (3.3)	11 (6) 13 (7) 13 (6) 14.5 (6.3)	24 (11.5) 23 (8) 24 (7) 24 (7.3)	16 (7.5) 17 (6) 17 (6) 17 (5)	11 (3) 10 (4) 10 (4) 10 (4)
*p*-value	0.021*	0.015*	0.639	0.076	0.007*	0.108	0.224	0.322
**Paying for prescriptions** Yes No	13.5 (5) 11 (7)	19 (6.3) 18 (5)	8.5 (5.5) 3 (0)	13.5 (4) 13 (4.3)	13 (6) 12 (6)	27 (8) 24 (7.3)	20 (6.3) 17 (5.3)	11 (3.3) 10 (4)
*p*-value	0.076	0.098	<0.001*	0.120	0.209	0.021*	0.025*	0.348
**Needing Help with using medicines** Yes No	12 (5) 11 (6)	20 (4) 18 (4)	3 (0) 3 (0)	14 (4) 12 (4)	14 (5) 12 (6)	25 (7) 23 (8)	19 (5) 16 (6)	10 (4) 9 (4)
*p*-value	<0.001*	<0.001*	0.213	0.001*	<0.001*	0.013*	<0.001*	0.027*

**^**Higher scores indicating a greater burden.

*p < 0.05.

Over three-fifths of respondents did not find difficulties in getting prescriptions from their doctors (70.8%; 95% CI: 66.1–75.0) or pharmacists (75.7%; 95% CI: 71.3–79.7), comfortable with the times for taking their medicines (71.9%; 95% CI: 67.4–76.1), concerned about forgetting to take their medicines (69.1%; 95% CI: 64.4–73.4), and it is easy for them to keep to their medicines routine (63.7%; 95% CI: 58.9–68.2). Over one-third (36.5%; 95% CI: 32.0–41.4) put a lot of planning and thought into using their medicines and 24.5% (95% CI: 20.6–29.0) found using their medicines difficult. Significant higher scores indicating more practical difficulties were found among those using oral solid dose and nonoral formulations, taking medicines 2 or more times daily, and needing help with using medicines (**Table 3**).

The cost of prescribed medicines was not burdensome for the vast majority of respondents (> 97.0%). Significant higher score indicating greater cost‐related burden was found among non-Kuwaiti residents who are paying prescription charges (**Table 3**). In relation to the side effects, 51.9%(95% CI: 47.0–56.7) of respondents strongly agreed/agreed that side effects were worse than the condition for which they used medicines. Over two-fifths of participants strongly agreed/agreed that side effects adversely affected their well-being (46.3%; 95% CI: 41.4–51.1), were bothersome (42.5%; 95% CI: 37.7–47.3), and interfered with their day-to-day life (42.2%;95% CI: 37.5–47.1). Significant higher scores indicating greater side effect‐related burden were found in respondents who had low-intermediate education, using five or more medications and needing help with using medicines (**Table 3**).

In relation to the perceived effectiveness of medicines, seven-in-ten respondents felt that their medicines are working (70.5%; 95% CI: 65.9–74.8), live up to their expectations (70.0%; 95% CI: 65.4–74.3), and prevented their conditions from getting worse (73.1%; 95% CI: 66.6–77.2). Over two-thirds of participants expressed satisfaction and reported the benefits. Significant higher scores indicating less satisfaction with medication effectiveness were found in respondents aged ≥75 years, males, employed, using oral solid dose and nonoral formulations, taking medicines two or more times daily, and needing help with using medicines. (**Table 3**).

The evaluation of attitudes/concerns about medicine revealed that 58.5%(95% CI: 53.6–63.2) of respondents felt that they need more information about their medicines, had concerns related to long‐term effects of using medicines (59.4%; 95% CI: 54.6–64.1), potential drug-drug interactions (57.3%; 95% CI: 52.4–62.1), and potential drug-nutrient interactions (54.5%; 95% CI: 49.5–59.3). Two-hundred forty-one (56.8%; 95% CI: 52.0–61.6) of respondents were worried about using several medicines at the same time and 52.8% (95% CI: 48.0–57.7) were concerned about being too reliant on their medications. One-quarter (25.2%; 95% CI: 21.2–29.7) of respondents would like more say in the brands of medicines used. Significant higher scores indicating more concerns about medicine use were found among non-Kuwaiti nationals who are paying prescription charges, using five or more medicines and needing help with using medicines (**Table 3**).

In relation to the impact/interference of medicines with day-to-day life, 57.5% (95% CI: 52.7–62.3) of participants indicated that their lives revolved around using their medicines. Interference with social or leisure activities indicated by 40.8% (95% CI: 36.1–45.7), with daily tasks by 33.3% (95% CI: 28.8–38.0), with social relationships by 27.8% (95% CI: 23.7–32.4), with driving by 22.2% (95% CI: 18.4–26.5), and with sexual life by 17.7% (95% CI: 14.2–21.7). Significant higher scores indicating greater interference with daily life were found among non-Kuwaiti residents who are paying prescription charges, using five or more medications, using oral solid dose and nonoral formulations, and needing help with using medicines (**Table 3**).

The assessment of the patients’ control/autonomy over their medication regimens identified that over half of respondents indicated limited empowerment to alter their medication regimens to suit their lifestyle, while 32.8% (95% CI: 28.4–37.5) felt able to change administration times, 31.4% (95% CI: 27.0–36.1) could change their medicines doses, and 27.1% (95% CI: 23.0–31.7) felt they had a choice in whether or not to use their medicines. Significant higher scores indicating a lack of control were found in patients using five or more medicines and needing help with using medicines (**Table 3**).

The median (IQR) overall LMQ-3 and VAS (IQR) scores were 112 (21) and 5 (4), respectively. The findings showed that the vast majority (n=413; 97.4%; 95% CI: 95.3–98.6) of the study population are perceived to suffer from minimum to moderate degrees of burden. **Table 4** presents the perceived MRB measured using LMQ-3 and VAS.

**Table 4:** Perceived medication-related burden measured using Living with Medicines Questionnaire version-3 (LMQ-3) and VAS (n=424).

Variable	*Range	Mean (SD)	Median (IQR)	Frequency (%, 95% CI)
**LMQ overall score** No burden at all Minimum burden Moderate burden High burden	(41–205) (41–73) (74–106) (107–139) (140–172)	110.4 (14.9)	112 (21.0)	3 (0.7; 0.2–2.2) 150 (35.4; 30.9–40.2) 263 (62.0, 57.2–66.6) 8 (1.9; 0.9–3.8)
Theme 1: Communication/relationships with healthcare professionals about medicines	(5–25)	11.8 (4.3)	11.0 (7.0)	
Theme 2: Practical Difficulties	(7–35)	18.7 (3.6)	18.0 (5.0)	
Theme 3: Cost-related burden	(3–15)	3.2 (1.2)	3.0 (0.0)	
Theme 4: Side-effects burden	(4–20)	12.9 (3.2)	13.0 (4.0)	
Theme 5: Perceived effectiveness of medicines	(6–30)	13.2 (4.5)	13.0 (6.0)	
Theme 6: Attitudes/concerns about medicine use	(7–35)	23.4 (5.4)	24.0 (7.0)	
Theme 7: Impact/interferences with day-to-day life	(6–30)	17.5 (4.3)	17.0 (6.0)	
Theme 8: Control/autonomy of medicine use	(3–15)	9.7 (2.6)	10.0 (4.0)	
**VAS: Global burden**	(0–10)	5.0 (2.3)	5.0 (4.0)	

Higher scores indicating a greater burden.

**Table 5** presents the effect of demographic and medication-related characteristics on LMQ-3 overall scores. LMQ-3 overall scores showed a significant trend toward higher perceived burden among respondents aged ≥ 75 years, males, non-Kuwaitis, residents in Al-Farwaniyah and Al-Jahra governorates, using oral solid dose and nonoral formulations, paying prescription charges, and needing support with using medicines (p<0.05). LMQ‐3 overall scores did not show any significant differences dependent on educational level, employment, number of medications, and dosing frequency (p>0.05).

**Table 5:** Effect of demographic and medication-related characteristics of respondents on Living with Medicines Questionnaire version-3 (LMQ-3) overall Scores (n=424).

Characteristics	LMQ overall Score		
	Median (IQR)	Mean (SD)	p-value
**Demographic characteristics**			
**Age (Years)** 65–74 ≥ 75	110 (22) 114 (20)	109.3 (15.1) 113.2 (14.1)	0.018*
**Gender** Male Female	114 (19) 110 (238)	112.8 (14.8) 108.3 (14.6)	0.004*
**Nationality** Kuwaiti nationals Non-Kuwaiti residents	111 (21) 125 (22.5)	109.7 (14.4) 125.3 (18.1)	<0.001*
**Education level** Low-Intermediate Education High Education	113 (23.3) 111 (18)	110.6 (15.6) 109.9 (13.7)	0.630
**Residence** Capital Hawalli Al-Farwaniyah Al-Ahmadi Al-Jahra Mubarak Al-Kabeer	110 (18) 110 (21.8) 116 (16.8) 109 (23) 121 (24) 110 (21.3)	108.5 (13) 108.6 (14.9) 116.8 (14.2) 105.8 (14.4) 119.3 (14.8) 109.4 (14.1)	<0.001*
**Employment status** Employed Unemployed/Retired	113 (11.3) 111 (22)	112.4 (10.4) 110.1 (15.4)	0.209
**Medication-related Characteristics**			
**Number of Medications** 1–4 ≥ 5	110 (24.3) 113 (18.3)	108.5 (15.4) 111.9 (14.3)	0.097
**Formulation used** Tablets/capsules Tablets/capsules + nonoral	110 (22) 115 (14)	109.1 (15.3) 114.6 (12.8)	0.002*
**Frequency of daily dose** Once Twice Three times More than three times	109 (27) 110.5 (23.3) 113 (19) 114.5 (20.5)	105.8 (16.6) 108.8 (15.4) 111.3 (13.8) 114.4 (15.6)	0.095
**Paying for prescriptions** Yes No	125 (22.5) 111 (21)	125.9 (17.3) 109.7 (14.4)	<0.001*
**Needing help with using medicines** Yes No	116 (16) 109 (21)	116.8 (12.4) 107.6 (15)	<0.001*

*p < 0.05.

**Table 6** shows the distribution of the responses to the ARMS items for assessing MA. The median (IQR) ARMS overall score was 20 (7.0) [mean (SD) 20.9 (4.9), range 12–42]. The results showed that 54.7% (n=232; 95% CI: 49.8–59.5) of respondents were nonadherent to their medications (had a score of ≥ 20 - low adherence) and 192 (45.3%: 95% CI: 40.5–50.2) had a score of < 20, which indicated a high level of MA. There was a significant positive correlation between LMQ-3 and ARMS scores (Spearman’s r= 0.404; p < 0.001). This relationship indicates that the higher the MRB the lower level of MA.

**Table 6:** Adherence to Refills and Medications Scale (ARMS) items assessing medication adherence among the respondents (n=424).

Item	None of the time	Some of the time	Most of the time	All of the time
	Frequency (%)			
1. How often do you forget to take your medicine?	112 (26.4)	257 (60.6)	48 (11.3)	7 (1.7)
2. How often do you decide not to take your medicine?	215 (50.7)	168 (39.6)	31 (7.3)	10 (2.4)
3. How often do you forget to get prescriptions filled?	253 (59.7)	126 (29.7)	40 (9.4)	5 (1.2)
4. How often do you run out of medicine?	215 (50.7)	156 (36.8)	44 (10.4)	9 (2.1)
5. How often do you skip a dose of your medicine before you go to the doctor?	152 (35.9)	207 (48.8)	53 (12.5)	12 (2.8)
6. How often do you miss taking your medicine when you feel better?	176 (41.5)	179 (42.2)	63 (14.9)	6 (1.4)
7. How often do you miss taking your medicine when you feel sick?	196 (46.2)	176 (41.5)	42 (9.9)	10 (2.4)
8. How often do you miss taking your medicine when you are careless?	222 (52.4)	155 (36.5)	39 (9.2)	8 (1.9)
9. How often do you change the dose of your medicines to suit your needs (like when you take more or less pills than you’re supposed to)?	205 (48.3)	166 (39.2)	45 (10.6)	8 (1.9)
10. How often do you forget to take your medicine when you are supposed to take it more than once a day?	98 (23.1)	215 (50.7)	96 (22.7)	15 (3.5)
11. How often do you put off refilling your medicines because they cost too much money?	413 (97.4)	5 (1.2)	2 (0.5)	4 (0.9)
12. How often do you plan ahead and refill your medicines before they run out?*	92 (21.7)	127 (30.0)	159 (37.5)	46 (10.8)

*Negatively worded statement was reverse coded for calculation of the score.

## Discussion

The present study appears to be the first attempt in Kuwait, and probably in the MENA region to assess MRB from the perspective of geriatric patients living with NCDs using the LMQ-3. Also, it is the second study in the MENA region to report MRB using the LMQ-3. The first study was conducted in Qatar among adult patients with diabetes, with or without other comorbidities (Zidan et al., 2018). In our current study, the vast majority (97.4%) of participants self-reported to suffer from a minimum (35.4%) to moderate (62.0%) degrees of burden. Over half (54.7%) of respondents were found to be nonadherent to their long-term medications based on ARMS overall score. Discrepancies in medication burden overall scores were identified regarding the demographic and medication-related characteristics. LMQ-3 overall scores revealed a significant trend toward higher perceived burden among respondents aged ≥ 75 years, males, non-Kuwaitis, residents in Al-Farwaniyah and Al-Jahra governorates, using oral solid dose and nonoral formulations, paying prescription charges, and needing support with using medicines (p <0.05). In the present study, LMQ overall scores did not show any significant differences related to the number of medications and dosing frequency. This indicates that the MRB for geriatric patients depends on various factors and that neither the number of medications nor dosing frequency alone is likely to help identify patients needing more support to cope with their medications. There was a significant positive correlation between LMQ-3 and ARMS scores indicating that the higher the MRB the lower level of MA. Moreover, the different types of issues perceived by respondents through the variation in the eight domains scores within the LMQ-3 demonstrated that MRB is multidimensional (Mohammed et al., 2016). Hence, the in-depth understanding regarding patient’s experience of using medications through the LMQ-3 could be useful in clinical practice to assist clinicians in identifying geriatric patients with MRB, who could benefit from individually tailored interventions targeting the contributing factors to help in reducing this burden (Krska et al., 2018). The current results could serve as an initial step in providing a baseline quantitative data of MRB and medication nonadherence among geriatric patients, which can be used for planning and evaluating any future interventions to reduce this burden and improve MA. Furthermore, the results from our study will support the usefulness of the LMQ-3 and allow for vital comparative work with prevailing and future similar studies in the MENA countries, and worldwide.

Few studies are currently available for comparison to the present study since assessing MRB using the LMQ-3 is relatively new. The current findings can be best compared to the recent studies performed in Qatar and England using the LMQ-3. However, these comparisons should be considered with caution due to the differences in the methodologies and health systems. Our present findings revealed that the vast majority of the study population reported experiencing minimal (35.4%) to moderate (62.0%) degrees of MRB, which is different from that reported in Qatar, minimal (66.8%) to moderate (24.1%) and in England, minimal (33.1%) to moderate (53.6%) degrees of burden, respectively (Zidan et al., 2018; Krska et al., 2019). This may be due to the differences among the study populations, both studies assessed MRB among patients aged ≥ 18 years. In the present study, the median (IQR) LMQ overall score of respondents was 112 (21), which is a moderate burden and greater than the minimum burden reported by the studies conducted in Qatar (95) and England (99.7) (Krska et al., 2018; Zidan et al., 2018). Also, the current findings revealed that the assessment of the global burden by the VAS score was 5, which is higher than score of 3 reported in Qatar (Zidan et al., 2018). These differences may be explained by the less number of geriatric patients included in both studies [Qatar, 37 (12.6%) patients and England, 277 patients (41.9%) compared with 424 patients in this study]. Also, the cultural differences between the populations may translate into different levels of perceiving a MRB (Zidan et al., 2018).

In relation to the effect of the demographic characteristics on MRB, the current results revealed that the median LMQ overall score among patients aged ≥75 years was significantly higher than those aged 65–74 years old. This finding is in contrast to the Qatari and English studies, which reported that LMQ overall scores were not strongly related to age (Katusiime et al., 2018; Zidan et al., 2018), and another English study that showed a significantly lower mean LMQ overall scores among elderly patients (≥ 65 years) compared with adult patients (18–64 years) and a nonsignificant difference among the older patient groups, 65–79 years vs. ≥ 80 years (Krska et al., 2018). The possible explanation for the high burden among respondents aged ≥75 years may be due to their significantly higher scores in two domains related to communication/relationships with healthcare professionals about medicines and perceived effectiveness of medicines, which revealed their poorer quality relationships with healthcare professionals and less satisfaction with medication effectiveness. Further qualitative research is needed to determine why those aged ≥75 years perceived high MRB. The median LMQ overall score for Kuwaiti nationals was significantly lower, revealing less MRB compared with non-Kuwaiti residents. In contrast, the median LMQ overall score for Qatari nationals was significantly higher, representing worse medication burden than non-Qataris (Zidan et al., 2018), and there was no significant difference dependent on ethnicity in the English study (Krska et al., 2018). The main explanation of high burden among non-Kuwaiti residents is the fact that they are paying prescription charges. Other possible explanations are their significant higher scores of burden related to attitudes/concerns about medicine use and impact/interference of medicines with day-to-day life compared with the Kuwaiti nationals, and the cultural differences that may translate into different levels of perceiving MRB. The present findings revealed significantly higher median LMQ overall score in males than females, which is in disagreement with previous studies that reported significantly higher levels of MRB among females (Sav et al., 2013; Sav et al., 2015; Zidan et al., 2018), and nonsignificant difference dependent on gender (Krska et al., 2018). This finding may be explained by the significantly higher scores of a burden that male respondents had regarding communication/relationships with healthcare professionals about medicines and perceived effectiveness of medicines. The presence of inconsistent information among several studies implies that assessments of MRB cannot be targeted toward certain patient characteristics or populations. The current results showed that patients resident in Al-Farwaniyah and Al-Jahra governorates perceived significantly higher levels of a burden than those in the other four residential areas. This could partly be explained by possible variations in the health status of the patients and clinical practice in that area. Future research is needed to better understand this regional variation.

In relation to the effect of the medicine-related characteristics on MRB, respondents using oral solid dose and nonoral formulations, paying prescription charges, and needing support with using medicines revealed significantly higher scores of medication burden. The high burden among those using nonoral formulations is in line with that reported in Qatar (Zidan et al., 2018) but in contrast to the English studies which did not find significant differences dependent on types of formulations (Krska et al., 2018; Krska et al., 2019). The possible explanations among respondents using oral solid dose and nonoral formulations such as injections, inhalers, and eye drops may be due to the significant higher burden they perceived concerning four domains, namely the communication/relationships with healthcare professionals about medicines, practical difficulties in the experience of using medicines, perceived effectiveness of medicines, and the impact/interference of medicines with day-to-day life. This highlights that the use of various medicine formulations with multiple instructions that need varying degrees of effort to use properly is burdensome to patients. The significant higher burden among participants paying for prescription charges is similar to that reported in England (Krska et al., 2018). This high burden could be explained by the significantly higher burden perceived by them regarding prescription cost, attitudes/concerns about medicine use, and the impact/interference of medicines with day-to-day life. Since all these respondents are non-Kuwaiti residents who are paying prescription charges, the cultural differences that may translate into different levels of perceiving MRB could not be excluded as an additional possible explanation. It has been reported that cost burden can lead to nonadherence, poor health outcomes, and impact on day-to-day lives, however, the cost of prescribed medications was not burdensome for more than 97.0% of the study participants, 95.8% were Kuwaiti nationals who do not pay a prescription charge. Respondents needing help with using medicines perceived a significantly higher burden compared with those who do not need help. This finding is in agreement with that reported in England (Krska et al., 2018), but in disagreement with that in Qatar where patients needing help with using medicines had lower levels of a burden than those who do not need help (Zidan et al., 2018). Respondents requiring help with using medicines perceived a significantly higher burden in all domains except cost. The need for support with using medicines may differ in nature, hence further research is needed to allow better understanding of how the need for help affects perceived burden (Krska et al., 2018). In the present study, LMQ overall scores did not show any significant differences dependent on the number of medication and frequency of doses, which are in line with the findings of the Qatari study (Zidan et al., 2018), but in contrast to the English study that showed a significant increase in the burden score with both increased number of medications and increased dosing frequency (Krska et al., 2018). Other English studies reported a nonsignificant association between the number of medications and increasing burden (Katusiime et al., 2018; Krska et al., 2019). This present finding demonstrated that from the patients’ perspectives, the medication-related overall burden is multifactorial and more than the number of medications to reach the threshold of being categorized as polypharmacy to contribute to medicines burden. Also, consistent with previous reports that some patients might be burdened by a few number of medications, whereas other patients do not perceive large numbers of medications to be burdensome (Tran et al., 2012; Mohammed et al., 2016). Despite that there was no significant difference in the overall burden scores dependent on the number of medications, participants taking five or more medications were found to have significantly higher scores among four domains, namely the side effects, attitudes/concerns toward medicine use, and impact/interference of medicines with day-to-day life. This underscores the need for targeting these specific problems during interventions. Respondents taking medicines more than three times daily perceived significantly higher burden related to communication/relationships with healthcare practitioners. While those taking medicines two or more times daily had significantly higher burden regarding practical difficulties in the experience of using medicines and perceived effectiveness of medicines. Interestingly, the domains with significantly higher burden among those taking five or more medicines and taking medicines frequently are different, which is in contrast to the previous findings indicated in England that interference with daily life and side effects increased with both number and dosing frequency, while practical difficulties increased only with dosing frequency (Krska et al., 2018) This underscores the importance of a better understanding about the patients’ lived experience of using medicines to assist the healthcare professionals in providing individually tailored interventions.

The current results showed that 54.7% of participants had a low level of MA (ARMS scores of ≥ 20), which is consistent with that reported by a study performed in Korea among elderly patients (52.5%) (Jin et al., 2016), but lower than that indicated in Qatar (84%) among patients aged ≥ 18 years with only 12.6% of them aged ≥ 65 years (Zidan et al., 2018). The prevalence that over half of participants were nonadherent to their long-term medicines is of specific concern as a factor potentially leading to poor clinical outcomes, resulting in rehospitalization, increased mortality, and increased healthcare expenditures (Sokol et al., 2005; Simpson et al., 2006). This finding highlights the need for improving MA among geriatric patients to get the optimal benefit from their prescribed medications. Furthermore, this study demonstrated a significant positive correlation between the scores of the perceived medication burden and the self-reported MA, which is in agreement with studies performed in Qatar and Australia (Tran et al., 2014; Zidan et al., 2018), and other qualitative studies (Krska et al., 2013; Sav et al., 2013). This finding indicates that patients with more MRB are less adherent to their medications, which can result in poor health outcomes and affect daily lives (Eton et al., 2012).

### Strengths and Limitations

The strengths of this study include (i) the high response rate, which could highlight the significance of this study in geriatric patients, and the length of time that they were keen to devote to completing the survey; (ii) the appropriate sample size and sampling methods to yield a representative data about the study population; thus, the current results can be generalized to apply to geriatric patients’ attending the primary care centers in Kuwait; and (iii) it fills in a gap in the limited current literature in developing countries and contributes to providing further information about the usefulness of the LMQ-3 and allows for vital comparative work with prevailing future similar studies in the MENA countries, and worldwide.

Certain potential limitations should be taken into account during the interpretation of the results from this study. The findings may not be representative of the geriatric population in Kuwait because the study sample was restricted to primary care centers, this under-represented those who are more sick and do not regularly attend the primary care settings. Hence to get more insight into the MRB and MA among the elderly people in Kuwait, further studies are needed among those attending the secondary and tertiary care settings, and those who are housebound and using prescription medicines delivery services. This type of research depends greatly on information provided by respondents and exposed to recall bias. Hence, there is a tendency for socially desirable behaviors to be over-reported or socially undesirable behaviors to be under-reported. The degree of truthful answers or verifying participants’ claims is not possible in this study, which was taken at face value. The anonymous completion of the survey and guaranteed confidentiality could minimize social desirability. A further limitation is the cross-sectional nature of the data that presented one point in time and, thus, does not reflect any amendments in participants’ lived experience about medicine use and adherence to medicines over time. In previous similar studies, the length of the questionnaire was reported as a potential barrier to completion; however, this was overcome in the present study by collecting the data by face-to-face structured interviews. Some of the participants commented on its length, whereas the majority commented on the items related to cost-burden, which were perceived to be of no direct relevance to most of them who are Kuwaiti nationals that do not pay a prescription charge. Additionally, a few questions related to interference with day-to-day life were perceived to be of nonrelevance to the culture or culturally unacceptable to be asked. We suggest the deletion of these questions from the LMQ-3 to improve its applicability in the clinical practice in Kuwait to identify those with a high burden who could potentially benefit from interventions. Despite these limitations, the current results provide crucial information for assessing MRB and MA among geriatric patients in Kuwait.

## Conclusions

The present findings indicate that the vast majority (97.4%) of geriatric patients suffer from minimum to moderate degrees of MRB and 54.7% were found to be nonadherent to their long-term medicines. The results demonstrate that the MRB for the study population is multidimensional, and neither the number of medicines or dosing frequency alone is likely to identify patients suffering from a MRB. The present findings underscore a call for a more active role that clinicians should have in understanding the patients’ lived experiences with the use of long-term medicines, to identify particular problems related to MRB that patients face, and provide patient-centered care through selection of treatment care plans that suit a patient’s life. This highlights the need for more dialog between the elderly people and clinicians regarding the various aspects of MRB. More understanding regarding the patient’s experience of using medications through the LMQ-3, could assist clinicians in providing individually tailored therapeutic care plan to achieve optimal patient outcomes. Such tailoring is recommended by international guidelines (Royal Pharmaceutical Society, 2013; National Institute for Health and Care Excellence, 2017). The key findings of this study underscore the need for multifaceted interventions that should incorporate the improvement of patient-provider partnership and patient-centered medicine optimization. Additionally, expanding pharmacists’ roles through the implementation of pharmaceutical care so they can assist physicians in quantifying the patient’s individual MRB and MA.

## Data Availability Statement

The raw data supporting the conclusions of this article will be made available by the corresponding author, without undue reservation.

## Ethics Statement

The studies involving human participants were reviewed and approved by Ministry of Health Ethical Committee, Kuwait. The patients/participants provided their written informed consent to participate in this study.

## Author Contributions

AAwa designed and supervised the study, performed the major part of data analysis, and wrote the manuscript. AAlh contributed to the design and pretesting of the study survey, data collection, entry, and interpretation. AAlb contributed to data collection, analysis and interpretation, and the discussion. AAlh and AAlb contributed in the review and editing of the manuscript.

## Conflict of Interest

The authors declare that the research was conducted in the absence of any commercial or financial relationships that could be construed as a potential conflict of interest.
